# Pterygoid Meningoencephalocele in Adulthood as a Possible Manifestation of Dysembryogenesis of the Sphenoid Bone: A Case Report

**DOI:** 10.1155/crot/2291894

**Published:** 2026-11-01

**Authors:** Lara Vehar, Jure Urbančič, Matej Vrabec, Domen Vozel

**Affiliations:** ^1^ Faculty of Medicine University of Ljubljana Vrazov Trg 2 Ljubljana 1000 Slovenia uni-lj.si; ^2^ Department of Otorhinolaryngology and Cervicofacial Surgery University Medical Centre Ljubljana Zaloška Cesta 2 Ljubljana 1000 Slovenia kclj.si; ^3^ Medilab Diagnostic Imaging Vodovodna Cesta 100 Ljubljana 1000 Slovenia

## Abstract

Meningoencephalocele is a rare defect of the skull base in which brain tissue and meninges herniate through a bony defect. Skull base meningoencephaloceles are rare, particularly those not associated with the sphenoid sinus or other aerated anatomical structures. Due to their nonspecific clinical presentation, recognition is often delayed. The aim of this paper is to present a rare case of a meningoencephalocele of the middle cranial base with an atypical localization and clinical presentation. We present a 66‐year‐old patient who was treated at a tertiary otorhinolaryngology center for a meningoencephalocele of the left middle cranial base in the region of the pterygoid process of the sphenoid bone. The disease manifested solely as a chronic, nonproductive cough resistant to pharmacological treatment, which for several years had been attributed to asthma. The patient had no history of craniofacial trauma, prior skull base surgery, or risk factors for elevated intracranial pressure. Clinical ENT and neurological examinations revealed no abnormalities. MRI and CT of the head, performed for other indications, later confirmed the presence of a meningoencephalocele. The patient was successfully treated with a transnasal endoscopic surgical approach with skull base reconstruction, after which the cough resolved immediately. The presented case of a pterygoid meningoencephalocele of the middle cranial base at an atypical location within the sphenoid bone demonstrates that meningoencephaloceles may present without involvement of aerated anatomical structures and without typical signs of cerebrospinal fluid (CSF) leakage. In our patient, CSF leakage manifested exclusively as chronic cough, leading to a delayed diagnosis. Given the absence of risk factors for intracranial hypertension, this case raises consideration of dysembryogenesis of the sphenoid bone as a possible cause of meningoencephalocele formation. This report highlights the importance of considering rare causes of persistent, unexplained symptoms and confirms the transnasal endoscopic surgical approach as an effective treatment method.

## 1. Introduction

Meningoencephalocele is a rare condition with an incidence of 1–3 per 10,000, characterized by herniation of brain tissue and meninges through a bony defect in the skull [1–3]. Such abnormalities may be congenital or acquired as a result of trauma, tumors, or developmental bony dehiscences [2, 3]. Chronic elevation of intracranial pressure may also contribute to the development of skull base meningoencephaloceles, particularly in idiopathic intracranial hypertension, which typically occurs in obese middle‐aged women [4–7]. Typical radiological signs of intracranial hypertension are often present, such as an empty sella, arachnoid granulations, and thinning of the skull base bones [8, 9]. The risk of developing meningoencephaloceles is higher in cases of extensive pneumatization of the sphenoid sinus, as prolonged elevation of intracranial pressure leads to thinning of the sinus bony walls, resulting in dehiscence and subsequent meningoencephalocele formation. An increased risk is also associated with extensive lateral pneumatization of the sphenoid sinus, which under conditions of long‐standing elevated intracranial pressure may lead to bony defects and consequently to skull base meningoencephaloceles [10]. A representative example is meningoencephaloceles of the lateral recess of the sphenoid sinus, which account for approximately 10% of all meningoencephaloceles [11]. Similarly, secondary intracranial hypertension due to tumors, hemorrhage, infection, or structural anomalies can increase pressure on the skull base and lead to the formation of meningoencephaloceles [12].

The typical clinical presentation of skull base meningoencephaloceles in the sinonasal region includes clear unilateral nasal discharge of cerebrospinal fluid (CSF) rhinorrhea, headache, and recurrent meningitis [13]. Patients may also exhibit symptoms of idiopathic intracranial hypertension, such as headache, visual disturbances, and pulsatile tinnitus [14], while signs of decreased intracranial pressure may also occur, including orthostatic headache and neck stiffness [15]. In some cases, epileptic seizures or other neurological deficits may develop when the herniated tissue consists of functional brain parenchyma [16]. In addition to a thorough otorhinolaryngological examination, including nasal and nasopharyngeal endoscopy, otomicroscopy, and audiological testing, an ophthalmological examination with funduscopy is required, as it may reveal papilledema as an early sign of intracranial hypertension [13]. The presence of CSF in nasal discharge can be objectively confirmed by detection of beta‐2 transferrin or beta‐trace protein [17]. Imaging studies are essential, particularly high‐resolution computed tomography (HRCT) [18] and magnetic resonance imaging (MRI) of the head [19] to localize the meningoencephalocele and identify signs of intracranial hypertension [20]. Treatment of skull base meningoencephaloceles in the sinonasal region is generally transnasal endoscopic surgery, aimed at removal of the herniated tissue and closure of the bony defect [21, 22].

Due to the relative paucity of literature on sphenoidal meningoencephaloceles, we present a case of a patient with a meningoencephalocele at an atypical site in the middle cranial base, in the region of the lateral wall of the sphenoid sinus, with an unusual clinical presentation of chronic cough that had been attributed to asthma. We draw attention to a rare form of pterygoid meningoencephalocele, in which early recognition may be challenging but is crucial for accurate diagnosis and appropriate management. The case is presented in accordance with the CARE guidelines [23].

## 2. Case Report

A 66‐year‐old male office worker was hospitalized at a tertiary referral ENT center for management of a meningoencephalocele of the left middle cranial fossa in the region of the base of the left pterygoid process (Figure 1).

**FIGURE 1:**
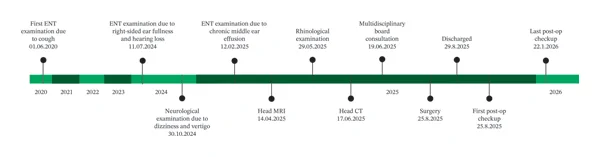
Timeline of the clinical management of the patient with a pterygoid meningoencephalocele.

In 2020, the patient was first referred for an ENT examination due to chronic cough in the setting of pharmacologically resistant asthma. The ENT examination revealed no abnormalities. In 2024, he was evaluated by a neurologist because of dizziness and vertigo. Ultrasound examination of the cervical arteries showed no abnormalities, and no additional imaging studies were performed at that time. In the same year, he was again evaluated by an otorhinolaryngologist due to a sensation of fullness and reduced hearing in the right ear, when right‐sided otitis media with effusion was diagnosed. A nasal glucocorticoid was prescribed, but the symptoms did not resolve. Due to persistent complaints, he was referred for brain MRI.

Apart from asthma, the patient had no other significant chronic diseases. In the past, he had undergone an appendectomy under general anesthesia. He denied any previous trauma or surgical procedures in the head and neck region. His regular therapy included an inhaled glucocorticoid (ciclesonide 160 μg) and pantoprazole 20 mg. As needed, he used short‐acting *β*_2_‐agonists (salbutamol and fenoterol) and a short‐acting anticholinergic agent (ipratropium bromide). No known addictions, allergies, colonizations, or hemostatic disorders were reported.

At admission, the patient showed no signs of neurological deficit or visual impairment. The facial skeleton was normally formed. On admission, he weighed 77 kg and was 188 cm tall (body mass index 21.7 kg/m^2^). ENT examination revealed no abnormalities other than the previously diagnosed right‐sided otitis media with effusion.

MRI of the brain and CT of the paranasal sinuses revealed a meningoencephalocele in the region of the left middle cranial fossa involving the sphenoid bone, lateral to the foramen ovale, extending between the medial and lateral plates of the pterygoid process, separating the pterygoid muscles, without contact with the paranasal sinuses (Figure 2). Imaging demonstrated a small dehiscence of the anterior part of the pterygoid process toward the pterygopalatine fossa (Figure 3) and an extensive dehiscence of the medial plate of the pterygoid process (Figure 2).

**FIGURE 2:**
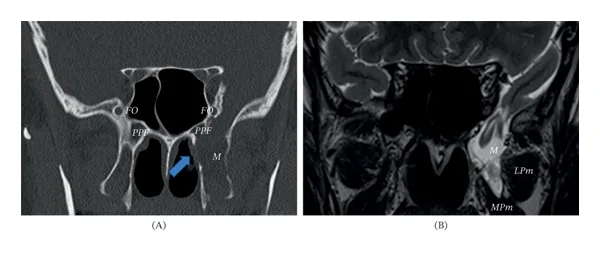
CT (A) and T2‐weighted MRI (B) of the paranasal sinuses in the coronal plane. An extensive dehiscence of the medial lamina of the pterygoid process toward the most posterior part of the nasal cavity, just anterior to the left choana (arrow), is visible. FO, foramen ovale; PPF, pterygopalatine fossa; M, meningoencephalocele; LPm, lateral pterygoid muscle; MPm, medial pterygoid muscle.

**FIGURE 3:**
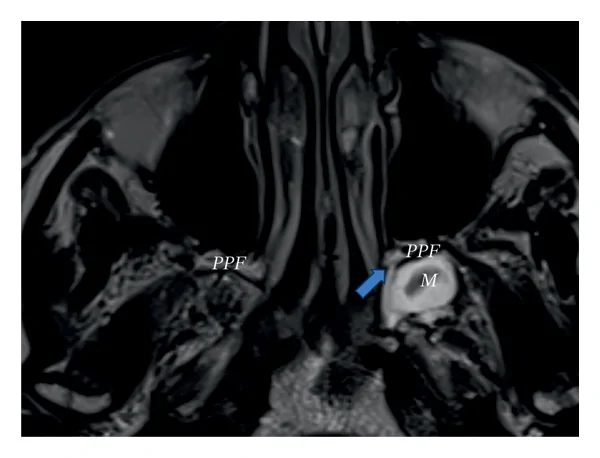
Axial T2‐weighted MRI just below the level of the sphenopalatine foramen. A T2‐hyperintense meningoencephalocele with herniated altered brain parenchyma (M) is visible. Dehiscence of the anterior part of the pterygoid process (arrow) toward the pterygopalatine fossa (PPF) is shown. M, meningoencephalocele; PPF, pterygopalatine fossa.

On completion of the Skull Base Inventory quality‐of‐life questionnaire prior to treatment, the patient scored 93 points [24].

Prior to surgery, a pulmonologist was consulted, who adjusted the inhaled corticosteroid therapy and introduced codeine in case of postoperative cough to prevent increases in intracranial pressure. Acetazolamide, other medications, or invasive measures for lowering intracranial pressure were not initiated.

On August 25, 2025, the patient underwent surgery under general anesthesia. A transnasal endoscopic left medial maxillectomy with resection of the nasolacrimal duct, left sphenoethmoidectomy, posterior septectomy, and left superior and partial inferior transpterygoid resection were performed to approach the meningoencephalocele via a binostril approach (Figure 4).

**FIGURE 4:**
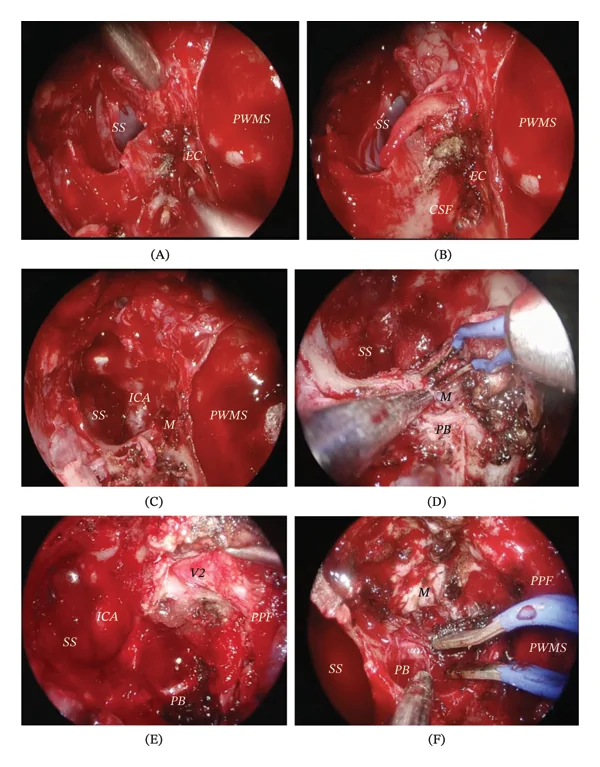
(A) Transnasal endoscopic view of the left sphenopalatine foramen; (B) visualization of cerebrospinal fluid (CSF) leakage from the sphenopalatine foramen posteriorly toward the nasopharynx; (C) initial view of the meningoencephalocele above the sphenopalatine foramen; (D) beginning of ablation of the meningoencephalocele using electrocautery; (E) visualization of the maxillary nerve and the cavity after removal of the meningoencephalocele; and (F) visualization of leveling of the meningoencephalocele with the adjacent middle cranial fossa skull base. SS, sphenoid sinus; EC, ethmoid crest; PWMS, posterior wall of the maxillary sinus; CSF, cerebrospinal fluid; ICA, paraclival internal carotid artery; M, meningoencephalocele; PB, pterygoid base; PPF, pterygopalatine fossa; V2, maxillary nerve entering the foramen rotundum.

During identification of the sphenopalatine artery, CSF leakage was observed. The left descending palatine neurovascular bundle and the Vidian nerve were identified and sacrificed, and the entire pterygopalatine fossa was lateralized to obtain the complete exposure of the meningoencephalocele in the pterygoid fossa. Upon pressure on the maxillary nerve, which was preserved, and the inferior orbital fissure, a marked transient bradycardia and asystole occurred.

The meningoencephalocele was localized using electromagnetic navigation (Fiagon, Cube Navigation System–VirtuEye HD, Neuendorfstrasse 23b, 16761 Hennigsdorf, Germany). Ablation of the meningoencephalocele was performed using electrocautery up to the level of the skull base.

Reconstruction was performed using autologous abdominal fat, TachoSil (Corza Medical, 2755 Canyon Boulevard, Boulder, CO 80302, USA), and a contralateral nasoseptal flap (Figure 5). The donor site of the flap was covered with a reverse nasoseptal flap. Additional support to the reconstruction was provided by placing fat into the resected pterygoid base toward the parapharyngeal space. The reconstructed area was further covered with multiple layers of TachoSil and DuraSeal (Integra LifeSciences Corporation, 311 Enterprise Drive, Plainsboro, NJ 08536, USA).

**FIGURE 5:**
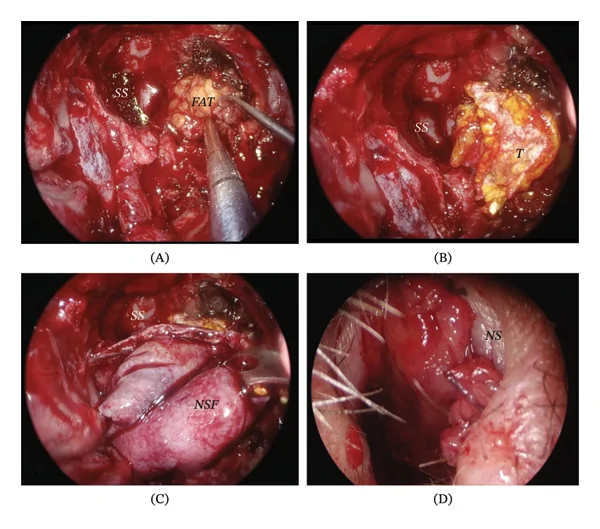
(A) Transnasal endoscopic view of reconstruction of the middle cranial fossa skull base using a fat graft; (B) placement of a heterologous resorbable hemostatic material (TachoSil); (C) placement of a right‐sided nasoseptal flap over the reconstruction layers; and (D) reconstruction of the donor site of the right nasoseptal flap using a reverse nasoseptal flap from the left side. SS, sphenoid sinus; T, TachoSil; NSF, nasoseptal flap; NS, nasal septum.

Subsequently, each nasal cavity was packed with Rapid Rhino (ArthroCare Corporation, 7000 West William Cannon Drive, Austin, TX 78735, USA). Intraoperatively, the patient received 1 g of intravenous tranexamic acid and 1.2 g of amoxicillin with clavulanic acid.

The postoperative course was uneventful. The patient remained cardiorespiratorily stable, with adequate analgesia, afebrile, and without CSF leakage, cough, or neurological deficits. Nasal packing was removed on the third postoperative day. The patient recovered according to the institutional protocol for patients undergoing skull base surgery [25]. On the fourth postoperative day, the patient was discharged to home care. Follow‐up examinations revealed no deviations from the expected postoperative course. At the most recent follow‐up visit, 12 months postoperatively, the patient reported no cough, and the nasal surgical sites were completely healed (Figure 6). The patient did not report any problems with dry eyes or sensory disturbances in the palate. The latter is expected to be postoperatively denervated, but the patient did not mention any problems related to this. Upon completion of the Skull Base Inventory quality‐of‐life questionnaire, the patient achieved a score of 87 points after treatment.

**FIGURE 6:**
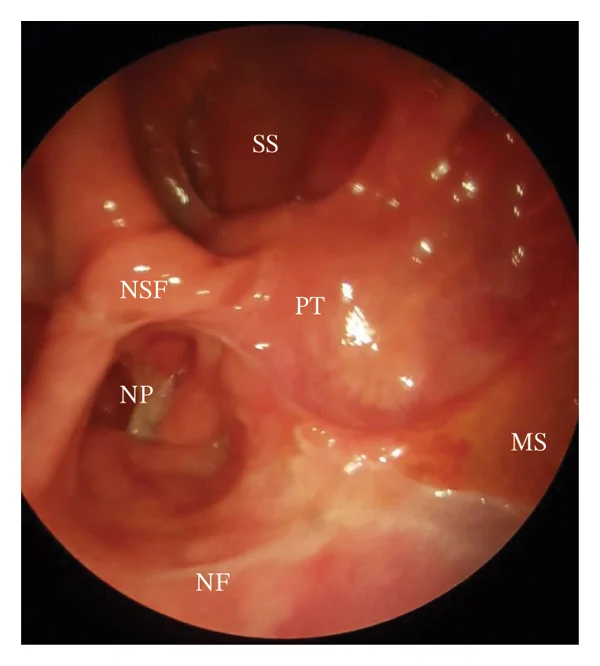
Endoscopic photograph of the postoperative state following a left‐sided transpterygoid approach, ablation of the meningoencephalocele, and reconstruction of the middle cranial fossa skull base. SS, sphenoid sinus; NSF, nasoseptal flap; NP, nasopharynx; NF, nasal floor; PT, pterygoid area; MS, maxillary sinus.

## 3. Discussion

This report presents a patient treated with transnasal endoscopic surgery for a meningoencephalocele of the middle cranial fossa located at an atypical site in the sphenoid bone, without involvement of aerated anatomical structures (nose, paranasal sinuses, and middle ear) and with CSF leakage manifesting as chronic cough resistant to pharmacological treatment. Based on its localization, this represents a pterygoid meningoencephalocele.

According to anatomical location, meningoencephaloceles are classified as occipital, sincipital (nasofrontal, nasoethmoidal, and naso‐orbital), and skull base types (spheno‐orbital, sphenomaxillary, transethmoidal, sphenoethmoidal, sphenonasopharyngeal, and basioccipital) [26, 27]. Meningoencephaloceles confined to the sphenoid sinus are particularly rare and constitute a distinct subtype [26, 27]. Among these, lesions of the lateral recess of the sphenoid sinus are clinically significant and well described. Their development was previously partly attributed to the presence of Sternberg’s canal [27–29]. This canal is an embryological remnant that is extremely rare in adults, and its pathophysiological role remains questionable [30]. Consequently, more recent studies suggest that chronic intracranial hypertension is a more likely pathogenetic mechanism, which, in combination with anatomical predisposition (e.g., extensive lateral pneumatization of the sphenoid sinus), leads to thinning of the skull base bone and the formation of bony dehiscences [11, 30]. This condition typically occurs in middle‐aged obese women with extensive pneumatization of the sphenoid sinus in the region of the lateral recesses, further supporting the association with elevated intracranial pressure [5–7].

In our patient, the disease was atypical in several respects and, to the best of our knowledge, has not previously been described in the literature. The meningoencephalocele was not located within the sphenoid sinus but adjacent to it, in the nonpneumatized portion of the sphenoid bone and in the soft tissues beneath the extracranial skull base. In contrast to conventional lateral sphenoid recess meningoencephaloceles, in which the bony defect communicates with the pneumatized lateral recess of the sphenoid sinus lateral to the foramen rotundum and Vidian nerve and posterior to the pterygopalatine fossa and pterygoid process [31], the skull‐base defect in our patient was located in the nonpneumatized sphenoid bone, lateral to the sphenoid sinus, foramen rotundum, and the Vidian canal, anterior to the foramen ovale and at the base of the pterygoid process (Figures 2 and 3). The meningoencephalocele extended through the pterygoid base into the pterygopalatine fossa, rather than through the lateral recess of the sphenoid sinus, allowing CSF to drain through the sphenopalatine foramen—an anatomical passage between the nasal cavity, nasopharynx, and pterygopalatine fossa—directly into the pharynx. This resulted in chronic cough as the primary symptom due to laryngeal irritation. The patient also had asthma, which was considered the cause of the pharmacologically resistant cough, contributing to the delayed diagnosis of the meningoencephalocele. A normal nasal and pharyngeal endoscopy further contributed to the delay. Testing for beta‐trace protein in nasal discharge was not performed, as imaging clearly confirmed the presence of a meningoencephalocele. Measurement of intracranial pressure, after consultation with a neurologist, was also not undertaken due to the absence of radiological and clinical signs of intracranial hypertension.

The mechanism of meningoencephalocele formation in our patient remains unknown. Given the absence of a lesion in the lateral recess of the sphenoid sinus, the degree of sphenoid sinus pneumatization can most likely be excluded as a causative or risk factor. The patient also lacked other risk factors for intracranial hypertension (e.g., obesity). Therefore, we may assume that the bony defect of the skull base lateral to the foramen rotundum and the Vidian canal, which led to the meningoencephalocele, developed before the complete maturation of the sphenoid sinus [32]. Because the firm soft tissues (including the pterygoid muscles and connective tissue) and bony structures (including the base of the pterygoid process) of the skull base provide substantial resistance to brain herniation, we hypothesize that in this patient the meningoencephalocele may have resulted from a disturbance of skull base embryogenesis between the 8th and 12th weeks of gestation, leading to subsequent herniation of brain parenchyma. More specifically, a developmental disturbance of the sphenoid cartilage could have resulted in impaired ossification of the medial pterygoid plate and the base of the pterygoid process, causing bony dehiscence and abnormal development of the pterygopalatine fossa into which the brain herniated. Dehiscence of the medial pterygoid plate allowed CSF to drain from the pterygopalatine fossa through the region of the sphenopalatine foramen into the nasopharynx [33, 34].

The choice of surgical approach may vary from surgeon to surgeon. Our surgical approach to the meningoencephalocele was not the decision of a single surgeon but rather that of the entire multidisciplinary skull base team, which includes at least otolaryngologists, neurosurgeons, and neuroradiologists. Meningoencephaloceles of the lateral recess of the sphenoid bone can also be treated with a less invasive approach that does not involve medial maxillectomy or a complete transpterygoid approach [35]; however, due to the need for a broader exposure of the lesion, a more extensive approach was required, which may also result in greater postoperative morbidity. Despite the resection of the greater palatine nerve, the patient did not experience any problems related to sensation in the palate. Although postoperative tearing in the left eye is likely reduced, the patient did not report any dry eye symptoms, which is consistent with the findings of the study by Wang et al. [36].

Compared with the available literature, this report offers consideration of an underlying developmental abnormality as a possible explanation for the unusual anatomical findings observed in our patient, regardless of the age at which the condition is diagnosed. It also provides insight into the atypical clinical presentation of a sinonasal meningoencephalocele and the resulting diagnostic delays despite appropriate evaluation by various specialists (e.g., otorhinolaryngologists and neurologists).

The main limitation of this report is the presentation of a single case with such a unique localization of the meningoencephalocele. Consequently, the hypothesis of dysembryogenesis is neither supported nor refuted by high‐level evidence. Nevertheless, this case may serve as a basis for further discussion and research into skull base embryology in relation to the causes of meningoencephalocele formation.

## 4. Conclusion

Pterygoid meningoencephalocele may present in adulthood in patients without identifiable risk factors, and the unusual anatomy observed in our patient raises the possibility of an underlying developmental abnormality of the sphenoid bone. It can manifest with a nonspecific clinical presentation and may mimic other diseases or conditions. This may lead to delayed treatment, which, in the case of sphenoid bone meningoencephaloceles, consists of transnasal endoscopic surgical management.

## Funding

This research received no specific grant or funding from any public, commercial, or not‐for‐profit organization.

## Consent

Written informed consent was obtained from the patient for publication of this case report.

## Conflicts of Interest

The authors declare no conflicts of interest.

## Data Availability

The data that support the findings of this study are available from the corresponding author upon reasonable request. The data are not publicly available due to privacy and ethical restrictions concerning patient information. All relevant data are included within the article.

## References

[1] Gisselsson L. , Intranasal Forms of Encephalomeningdcele, Acta Oto-Laryngologica. (1947) 35, no. 5-6, 519–531, 10.3109/00016484709123765.

[2] Karch S. B. and Urich H. , Occipital Encephalocele: a Morphological Study, Journal of the Neurological Sciences. (1972) 15, 89–112, 10.1016/0022-510X(72)90123-2. 5008718

[3] Leck I. , Changes in the Incidence of Neural-Tube Defects, The Lancet. (1966) 288, 791–793, 10.1016/S0140-6736(66)90383-7. 4162336

[4] Wall M. , Idiopathic Intracranial Hypertension, Neurologic Clinics. (2010) 28, 593–617, 10.1016/j.ncl.2010.03.003. PMC2908600 20637991

[5] Wise S. K. and Schlosser R. J. , Evaluation of Spontaneous Nasal Cerebrospinal Fluid Leaks, Current Opinion in Otolaryngology & Head and Neck Surgery. (2007) 15, 28–34, 10.1097/MOO.0b013e328011bc76. 17211180

[6] Schlosser R. J. , Woodworth B. A. , Wilensky E. M. , Grady M. S. , and Bolger W. E. , Spontaneous Cerebrospinal Fluid Leaks: a Variant of Benign Intracranial Hypertension, Annals of Otology, Rhinology & Laryngology. (2006) 115, 495–500, 10.1177/000348940611500703. 16900803

[7] Szewka A. J. , Bruce B. B. , Newman N. J. , and Biousse V. , Idiopathic Intracranial Hypertension: Relation Between Obesity and Visual Outcomes, Journal of Neuro-Ophthalmology. (2013) 33, 4–8, 10.1097/WNO.0b013e31823f852d. PMC3323689 22217456

[8] Silver R. I. , Moonis G. , Schlosser R. J. , Bolger W. E. , and Loevner L. A. , Radiographic Signs of Elevated Intracranial Pressure in Idiopathic Cerebrospinal Fluid Leaks: A Possible Presentation of Idiopathic Intracranial Hypertension, American Journal of Rhinology. (2007) 21, 257–261, 10.2500/ajr.2007.21.3026. 17621805

[9] Schlosser R. J. and Bolger W. E. , Significance of Empty Sella in Cerebrospinal Fluid Leaks, Otolaryngology-Head and Neck Surgery. (2003) 128, 32–38, 10.1067/mhn.2003.43. 12574756

[10] Lai S. Y. , Kennedy D. W. , and Bolger W. E. , Sphenoid Encephaloceles: Disease Management and Identification of Lesions Within the Lateral Recess of the Sphenoid Sinus, The Laryngoscope. (2002) 112, 1800–1805, 10.1097/00005537-200210000-00018. 12368619

[11] Hanz S. Z. , Arko L. , Schmidt F. et al., Low Incidence of True Sternberg’s Canal Defects Among Lateral Sphenoid Sinus Encephaloceles, Acta Neurochirurgica. (2020) 162, 2413–2420, 10.1007/s00701-020-04329-2. 32372133

[12] Poczos P. , Cihlo M. , Zadrobílek K. et al., The Need for Emphasis on Intracranial Hypertension Management in Spontaneous Skull Base Meningoencephaloceles, World Neurosurgery. (2023) 172, e574–e580, 10.1016/j.wneu.2023.01.083. 36716857

[13] Georgalas C. , Oostra A. , Ahmed S. et al., International Consensus Statement: Spontaneous Cerebrospinal Fluid Rhinorrhea, International Forum of Allergy & Rhinology. (2021) 11, 794–803, 10.1002/alr.22704. 33099888

[14] Camras L. R. , Ecanow J. S. , and Abood C. A. , Spontaneous Cerebrospinal Fluid Rhinorrhea in a Patient with Pseudotumor cerebri, Journal of Neuroimaging. (1998) 8, 41–42, 10.1111/jon19988141. 9442590

[15] Rudnick E. and Sismanis A. , Pulsatile Tinnitus and Spontaneous Cerebrospinal Fluid Rhinorrhea: Indicators of Benign Intracranial Hypertension Syndrome, Otology & Neurotology. (2005) 26, 166–168, 10.1097/00129492-200503000-00006. 15793399

[16] Alonso R. C. , De La Peña M. J. , Caicoya A. G. , Rodriguez M. R. , Moreno E. A. , and De Vega Fernandez V. M. , Spontaneous Skull Base Meningoencephaloceles and Cerebrospinal Fluid Fistulas, RadioGraphics. (2013) 33, 553–570, 10.1148/rg.332125028. 23479713

[17] Oberascher G. and Arrer E. , [Initial Clinical Experiences With Beta 2-Transferrin in Oto- and Rhinorrhea], HNO. (1986) 34, 151–155. 3710843

[18] Lund V.J. , Savy L. , Lloyd G. , and Howard D. , Optimum Imaging and Diagnosis of Cerebrospinal Fluid Rhinorrhoea, Journal of Laryngology & Otology. (2000) 114, 10.1258/0022215001904572. 11177378

[19] Oakley G. M. , Alt J. A. , Schlosser R. J. , Harvey R. J. , and Orlandi R. R. , Diagnosis of Cerebrospinal Fluid Rhinorrhea: an Evidence‐Based Review with Recommendations, International Forum of Allergy & Rhinology. (2016) 6, 8–16, 10.1002/alr.21637. 26370330

[20] Psaltis A. J. , Overton L. J. , Thomas W. W. , Fox N. F. , Banks C. A. , and Schlosser R. J. , Differences in Skull Base Thickness in Patients with Spontaneous Cerebrospinal Fluid Leaks, American Journal of Rhinology & Allergy. (2014) 28, e73–e79, 10.2500/ajra.2014.28.4002. 24717891

[21] Umemoto S. , Matsunaga T. , Momii Y. , Fujiki M. , and Hirano T. , Spontaneous Lateral Sphenoid Sinus Meningocele Presenting with Recurrent Meningitis: Surgical Resolution via an Endoscopic Endonasal Transpterygoid Approach, Cureus. (2025) 10.7759/cureus.89962. PMC12426875 40951134

[22] Yildirim A. E. , Divanlioglu D. , Cetinalp N. E. , and Belen A. D. , Endoscopic Endonasal Repair of Spontaneous Sphenoid Sinus Lateral Wall Meningocele Presenting With Cerebrospinal Fluid Leak, Journal of Neurosciences in Rural Practice. (2014) 5, 168–170, 10.4103/0976-3147.131668. PMC4064186 24966559

[23] Riley D. S. , Barber M. S. , Kienle G. S. et al., CARE Guidelines for Case Reports: Explanation and Elaboration Document, Journal of Clinical Epidemiology. (2017) 89, 218–235, 10.1016/j.jclinepi.2017.04.026. 28529185

[24] Vozel D. , Urbancic J. , Battelino S. et al., Quality of Life in Slovenian Patients with Skull Base Tumours: Cross-Cultural Adaptation and Validation of a Slovenian Skull Base Inventory, Radiology and Oncology. (2026) 10.2478/raon-2026-0007. PMC13012383 41560283

[25] Vozel D. and Urbančič J. , Postoperative Management Following Endoscopic Skull Base Surgery, Current Opinion in Otolaryngology & Head and Neck Surgery. (2025) 33, 7–12, 10.1097/MOO.0000000000001010. 39365142

[26] Buchfelder M. , Fahlbusch R. , Huk W. J. , and Thierauf P. , Intrasphenoidal Encephaloceles?A Clinical Entity, Acta Neurochirurgica. (1987) 89, 10–15, 10.1007/BF01406661. 3324649

[27] You W. , Lin Z.-R. , Wang Y.-Z. et al., Analysis of the Causes and Experience in the Diagnosis and Treatment ofMeningocele Caused by Sternberg’s Canal of the Sphenoid Sinus: Twocase Reports and a Review of the Literature, CMIR. (2023) 19, 10.2174/1573405619666230206103036. 36748216

[28] Tomazic P. V. and Stammberger H. , Spontaneous CSF-Leaks and Meningoencephaloceles in Sphenoid Sinus by Persisting Sternberg’s Canal, Rhinology. (2009) 47, 369–374, 10.4193/Rhin08.236. 19936360

[29] Schick B. , Prescher A. , and Brors D. , Sternberg’s Canal-Cause of Congenital Sphenoidal Meningocele, European Archives of Oto-Rhino-Laryngology. (2000) 257, 430–432, 10.1007/s004050000235. 11073192

[30] Barañano C. F. , Cure J. , Palmer J. N. , and Woodworth B. A. , Sternberg’s Canal: Fact or Fiction?, American Journal of Rhinology & Allergy. (2009) 23, 167–171, 10.2500/ajra.2009.23.3290. 19401043

[31] Alokby G. , Albathi A. , Alshurafa Z. , and AlQahtani A. , Endoscopic Endonasal Repair of a Temporal Lobe Meningoencephalocele in the Pterygoid Fossa: a Case Report and Literature Review, International Journal of Surgery Case Reports. (2021) 83, 10.1016/j.ijscr.2021.105963. PMC8164042 34022760

[32] Aksakal C. , Aktı S. , Çeker M. E. , Subaşı Aksakal B. , Sapmaz E. , and Gökçe E. , Development of the Sphenoid Sinus from Newborn to Age 18: a Computed Tomography Imaging Analysis, International Journal of Pediatric Otorhinolaryngology. (2022) 162, 10.1016/j.ijporl.2022.111327. 36202021

[33] Bonneville J. F. and Dietemann J. L. , Embryology of the Sellar Region, Radiology of the Sella Turcica. (1981) Springer Berlin Heidelberg, Berlin, Heidelberg, 1–8, 10.1007/978-3-642-67786-1_1.

[34] Triantafyllou G. and Piagkou M. , The Developing Human Sphenoid Bone: Linking Embryological Development to Adult Morphology, Biology. (2025) 14, 10.3390/biology14081090. PMC12384024 40906422

[35] Sreenath S. B. , Tang D. M. , Ting J. Y. et al., Modified Transpterygoid Approach to Sphenoid Meningoencephaloceles: a Shorter Run for a Longer Slide, The Laryngoscope. (2021) 131, 2224–2230, 10.1002/lary.29672. 34096616

[36] Wang E. W. , Gardner P. A. , Fraser S. , Stefko S. T. , Fernandez-Miranda J. C. , and Snyderman C. H. , Reduced Tearing with Stable Quality of Life After Vidian Neurectomy: a Prospective Controlled Trial, The Laryngoscope. (2021) 131, 1487–1491, 10.1002/lary.29287. 33247625

